# Cortical and white matter correlates of language‐learning aptitudes

**DOI:** 10.1002/hbm.25598

**Published:** 2021-07-20

**Authors:** Mikael Novén, Hampus Olsson, Gunther Helms, Merle Horne, Markus Nilsson, Mikael Roll

**Affiliations:** ^1^ Department of Linguistics and Phonetics Lund University Lund Sweden; ^2^ Department of Clinical Sciences Lund, Medical Radiation Physics Lund University Lund Sweden; ^3^ Department of Clinical Sciences Lund Radiology, Lund University Lund Sweden

**Keywords:** cortical morphometry, cortical surface area, cortical thickness, dwMRI, language‐learning aptitude, ultra‐high field

## Abstract

People learn new languages with varying degrees of success but what are the neuroanatomical correlates of the difference in language‐learning aptitude? In this study, we set out to investigate how differences in cortical morphology and white matter microstructure correlate with aptitudes for vocabulary learning, phonetic memory, and grammatical inferencing as measured by the first‐language neutral LLAMA test battery. We used ultra‐high field (7T) magnetic resonance imaging to estimate the cortical thickness and surface area from sub‐millimeter resolved image volumes. Further, diffusion kurtosis imaging was used to map diffusion properties related to the tissue microstructure from known language‐related white matter tracts. We found a correlation between cortical surface area in the left posterior‐inferior precuneus and vocabulary learning aptitude, possibly indicating a greater predisposition for storing word‐figure associations. Moreover, we report negative correlations between scores for phonetic memory and axial kurtosis in left arcuate fasciculus as well as mean kurtosis, axial kurtosis, and radial kurtosis of the left superior longitudinal fasciculus III, which are tracts connecting cortical areas important for phonological working memory.

## Graphical abstract

We found that cortical surface area in a left posterior medial cluster correlated with vocabulary learning aptitude, possibly reflecting a greater declarative memory storing capacity for linguistically relevant word‐figure associations. Moreover, the kurtosis parameters in known dorsal language processing stream tracts correlated negatively with phonetic memory. This indicates an impact from the coherency and homogeneity of white matter tracts connecting well‐known cortical areas responsible for phonological storage on phonetic working memory.

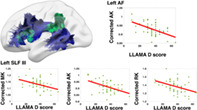

## 1 INTRODUCTION

The study of what makes a good language learner has been a growing research field since the 1950's (Carroll & Sapon, 1959). The need and benefit for people to learn new languages far into adulthood has only grown with globalization, which further spurs the interest to understand the nature of language‐learning aptitude. Language‐learning aptitude is a stable trait within individuals (Granena, 2013) that predicts how well people can learn a foreign language (Li, 2014). Brain structure has been shown to correlate with various aspects of language‐related performance, ranging from low‐level acoustic processing to executive control of languages in terms of fluency and speech‐in‐noise processing (Golestani, 2012). Heschl's gyrus (HG) even differs in shape between expert phoneticians and nonexperts, probably already before phonetic training (Golestani, Price, & Scott, 2011). Research into the associations between language‐learning aptitude and cortical morphology has found that the grey matter volume or shape of HG affects the ability to learn Mandarin word tones (Wong et al., 2008), performance on a speech imitation task including novel phonological contrasts (Turker, Reiterer, Seither‐Preisler, & Schneider, 2017), and general language‐learning aptitude (Turker, Reiterer, Schneider, & Seither‐Preisler, 2019). In addition, cortical thickness of anterior insula correlates with the aptitude for learning to discriminate new speech sounds in bilinguals but not monolinguals (Rodriguez, Archila‐Suerte, Vaughn, Chiarello, & Hernandez, 2018). Furthermore, Novén, Schremm, Nilsson, Horne, and Roll (2019) found the cortical thickness of Brodmann area (BA) 45 in the left inferior frontal gyrus (IFG) to correlate with grammatical inferencing ability. Studies including measures of white matter microstructure have instead observed associations between artificial grammar learning ability and the structure of connections to the left IFG (Flöel, de Vries, Scholz, Breitenstein, & Johansen‐Berg, 2009) and structure of right‐hemispheric white matter and successful learning of Mandarin (Qi, Han, Garel, San Chen, & Gabrieli, 2015). Moreover, white matter microstructure in frontal networks predicts language learning proficiency in conscript interpreters (Mårtensson et al., 2020). However, previous studies have not specifically related to language‐learning aptitude theory, which strives toward understanding what cognitive processes are involved in making a good language learner (Wen, Biedroń, & Skehan, 2016). Thus, language‐learning aptitude has most often been inferred by learning outcomes in a classroom environment (Mårtensson et al., 2020; Qi et al., 2015) or in learning isolated language elements (Flöel et al., 2009; Rodriguez et al., 2018; Turker et al., 2017; Wong et al., 2008). In fact, only two studies we know of, apart from our previous work (Novén et al., 2019), have examined brain structural correlates of behavioral measures rooted in language‐learning aptitude theory (Turker et al., 2019; Xiang et al., 2012). With regards to the parameters of brain structure studied, some have only investigated cortical morphology in predefined regions of interest (ROIs; Rodriguez et al., 2018; Turker et al., 2017, 2019; Wong et al., 2008), while others have been limited in spatial resolution (Novén et al., 2019). Further, cortical surface area has been overlooked as a potential correlate to language‐learning aptitude. This is despite the fact that cortical thickness and cortical surface area are independent measures of cortical morphology (Meyer, Liem, Hirsiger, Jäncke, & Hänggi, 2013; Panizzon et al., 2009; Vuoksimaa et al., 2015). Also, only standard diffusion tensor imaging (DTI) has previously been used to examine tissue microstructure correlates of language‐learning aptitude (Flöel et al., 2009; Mårtensson et al., 2020; Qi et al., 2015; Xiang et al., 2012). Using instead diffusion kurtosis imaging (DKI), it is possible to capture more detailed information about the tissue microstructure (Jensen & Helpern, 2010). Building on the work in Novén et al. (2019), the present study investigates the cortical morphometry, that is, the thickness and surface area of the cortex, at sub‐millimeter resolution. Further, DKI is used to inspect the microstructure of language‐relevant white matter tracts. Language‐learning aptitude is measured by the LLAMA test battery (Meara, 2005), based on the work of Carroll (1962). The refined techniques allow for measuring anatomical correlates of language‐learning aptitude in more detail than previous studies.

### 1.1 LLAMA test battery and brain structure

Language‐learning aptitude can be assessed using the first language‐independent, computer‐based LLAMA test battery (Meara, 2005). It has four sub‐tests assessing different aptitude components: written vocabulary (LLAMA B), phonetic memory (LLAMA D), sound‐symbol correspondence (LLAMA E), and grammatical inferencing (LLAMA F). The brain structure correlates of LLAMA test scores have been investigated in three studies, described below. Grey matter volume in the right, but not left, HG correlates with LLAMA scores from all sub‐tests except phonetic memory in children, 10–16 years of age (Turker et al., 2019). Sound‐symbol correspondence aptitude has been shown to correlate with the fractional anisotropy (FA) in a tract connecting BA45 in each hemisphere (Xiang et al., 2012). Moreover, vocabulary learning aptitude has been observed to correlate with a difference between FA in left and right tracts between BA47 and the parietal lobe (Xiang et al., 2012). We have previously seen a correlation between cortical thickness in left BA45 and medial frontal gyrus and grammatical inferencing aptitude (Novén et al., 2019). This is in line with the finding that the left‐lateralization of FA in the connection between BA 45 and the posterior temporal lobe as well as the sum of the number of streamlines between BA6 and the posterior temporal lobe from both hemispheres correlates with grammatical inferencing aptitude (Xiang et al., 2012). Taken together, previous studies have reported correlates between LLAMA test scores and cortical volume in a predefined ROI (Turker et al., 2019) or using data of lower resolution for cortical thickness estimation (Novén et al., 2019) and no studies have investigated possible correlations with cortical surface area. Tissue microstructure in white matter tracts is associated with language‐learning aptitude but has only been investigated using standard DTI (F. C. K. Wong, Chandrasekaran, Garibaldi, & Wong, 2011; Xiang et al., 2012) and construction of white matter tracts through probabilistic tracking from seed regions derived from functional experiments (Xiang et al., 2012). This method runs the risk of including nonanatomical white matter tracts (Schilling et al., 2019).

### 1.2 White matter tract segmentation

Four anatomical white matter tracts are essential for language processing and could be relevant for language‐learning aptitudes: the arcuate fasciculus (AF), subcomponent three of the superior longitudinal fasciculus (SLF III), the uncinate fasciculus (UF), and the inferior frontal‐occipital fasciculus (IFOF). AF connects the IFG and the middle frontal gyrus with the posterior superior temporal gyrus as well as the temporal occipital transition region (Catani, Jones, & ffytche, 2005; Makris et al., 2004). The left AF is used for mapping sound features to articulatory representations (Saur et al., 2008; Wong et al., 2011) and complex syntactic processing (Friederici & Gierhan, 2013). SLF links the frontal lobe with the lateral occipital and temporal lobes and is divided into three subcomponents, I–III (Makris et al., 2004). SLF III connects the supramarginal gyrus with the prefrontal and ventral premotor cortices and is the SLF subcomponent most implicated in neurolinguistic research due to its involvement in the ability to repeat speech (Friederici & Gierhan, 2013; Saur et al., 2008). UF joins the anterior temporal lobe and the frontal lobe (Catani & Thiebaut de Schotten, 2008). IFOF extends between the occipital lobe and the orbito‐ and inferior frontal cortices (Catani & Thiebaut de Schotten, 2008). Both UF and IFOF are suggested to be important for being able to map sounds to meaning (Wong et al., 2011). UF is further involved in the construction of short syntactic phrases (Friederici & Gierhan, 2013).

From diffusion‐weighted MRI data, it is possible to compute parameters that describe white matter tissue microstructure. Detailed descriptions and derivations of the parameters can be found in for example, Pierpaoli, Jezzard, Basser, Barnett, and Di Chiro (1996) and Jensen and Helpern (2010) for DTI and DKI, respectively. Put simply, spontaneous movement of water molecules within tissues occurs at different rates (diffusivities) along different spatial directions, depending on the microstructure of the tissue. DTI yields measures of the diffusivities along different directions, and thus an understanding of the anisotropy (i.e., how much easier it is for water to move along the most unrestricted direction than along the most restricted direction) of the tissue. However, DTI is based on the assumption that the diffusion properties are Gaussian, meaning that, along a given direction, the tissue is assumed to be homogenous (i.e., no variability in the obstruction of the water movement). Important DTI parameters are the mean diffusivity (MD), the mean apparent diffusivity across all diffusion encoding angles, and axial and radial diffusivity (AD and RD), the diffusivities along and perpendicular to the direction of greatest diffusivity. FA quantifies how much greater AD is than RD and ranges from 0 (water can move as easily in all directions) to 1 (water can only move along one direction). However, if complex tissues and macromolecules restrict diffusion, the water displacement profile becomes less Gaussian. The shape of the displacement profile is captured by the kurtosis (the standardized and normalized fourth central moment of the displacement probability distribution function). Axial and radial kurtoses (AK and RK) describe the diffusion kurtoses along or perpendicular to the principal diffusion direction while mean kurtosis (MK) is the mean kurtosis across all encoding directions. The diffusivity parameters (MD, AD, RD, and FA) are thus measures of the voxel‐average diffusion profile while the diffusion kurtosis parameters (MK, AK, and RK) give an estimation of the sub‐voxel structure of the tissue. It is also possible to use diffusion‐weighted MRI to construct white matter tracts through probabilistic tracking from seed regions derived from functional experiments (Xiang et al., 2012), but this is at the risk of including nonanatomical, that is, false, white matter tracts (Schilling et al., 2019). Therefore, we used a white matter segmentation software tool (TractSeg) to automatically segment anatomically relevant, language‐related tracts (Wasserthal, Neher, Hirjak, & Maier‐Hein, 2019; Wasserthal, Neher, & Maier‐Hein, 2018a, 2018b).

Taken together, the tissue microstructure of the language‐related tracts could be a source for or reflection of language‐learning aptitude as the tracts structurally connect, that is, allow for signal transport between cortical areas important for language processing. Therefore, we extracted mean diffusion parameter values, reflecting the microstructure of the tissue, from mentioned tracts and tested their correlations with LLAMA test scores.

### 1.3 The present study

The present study investigated the neuroanatomical correlates of language‐learning aptitude using the LLAMA tests and ultra‐high field MRI. The benefits of using ultra‐high field MRI lie in the increased signal‐to‐noise ratio and consecutive tissue contrast (Duyn, 2012) allowing for high resolutions, limiting partial volume effects, and yielding more effective tissue segmentations from T1‐weighted image volumes (Zaretskaya, Fischl, Reuter, Renvall, & Polimeni, 2018). This study contributes to the understanding of the structural neural correlates of language‐learning aptitude by using higher spatial resolution than in previous studies (Wong et al., 2011; Xiang et al., 2012), as a basis for cortical morphometry. Furthermore, utilizing DKI allows for the calculation of more detailed tissue microstructure parameters of relevant white matter tracts as compared to standard DTI.

## 2 METHODS

### 2.1 Participants

Fifty‐seven university students (15 men, 42 women) growing up in monolingual families (35 German and 22 Swedish) were recruited for this study. Mean age was 22.7 years (range = 20–27 years). No participant had any history of psychiatric disorders. All participants were right‐handed as defined as a minimum of +25 in the Edinburgh handedness index (Oldfield, 1971) and had vision that was normal or corrected to normal. To ensure normal hearing (necessary for the phonetic memory subtest), a minimal hearing threshold of <20 dB for pure tones of 250, 500, 1,000, 2,000, 4,000, and 8,000 Hz frequency was required to participate. Hearing thresholds were measured using fixed‐frequency Békésy audiometry in the same way and using the same equipment as in Novén et al. (2019).

Participants were characterized concerning their fluid intelligence, working memory capacity, and musical sophistication. Fluid intelligence was assessed by a short‐form of the Raven's matrices (Raven, 2000) described in Mårtensson and Lövdén (2011). Participants were given 10 min to complete 18 matrices by selecting the missing ninth pattern based on eight given patterns. Scores were equal to the number of correctly chosen patterns. Participants' working memory capacity was measured using an automated version of the operation span test (Unsworth, Heitz, Schrock, & Engle, 2005). Participants were required to solve arithmetic problems while remembering series of letters. The final score is the total number of correctly recalled letters. Musical sophistication was judged using the Goldsmith musical sophistication index (Gold‐MSI; Müllensiefen, Gingras, Musil, & Stewart, 2014). The local ethics board approved the study and all participants gave written consent prior to the experiment.

### 2.2 LLAMA tests

To measure participants' language‐learning aptitude, three of the four LLAMA tests were administered: The vocabulary (LLAMA B) subtest, the phonetic memory (LLAMA D) subtest, and the grammatical inferencing (LLAMA F) subtest. The fourth subtest, LLAMA E, focusing on sound‐symbol correspondence, was left out.

In the LLAMA B subtest, participants were shown 20 cartoon objects and were given the written name of each object when clicking on them with the computer mouse. Each participant was given 2 min to learn as many names as possible. In the test phase, each name was given and the participant was instructed to click the corresponding object on the screen. Feedback was given on the validity of the participant's choice for each answer. LLAMA B can thus be considered to test proficiency in identifying written word‐picture meaning correspondences.

The LLAMA D subtest started with the participants being asked to listen to a short set of spoken foreign words. Immediately afterward, either words found in the initial phase of the test or new words were played to the participant who was asked to indicate if the word was part of the initial set of words or not. This test can be assumed to assess phonetic memory capacity.

The LLAMA F subtest consists of 20 image‐sentence pairs consisting of pictures of stylized figures performing different actions. The images are associated with sentences consisting of strings of written pseudowords. The pictures and word strings were shown to participants as they clicked on boxes on the screen in a 5‐min training phase. They were told that they should use the time to learn as much as possible about the language used in the sentences to describe the images. Taking written notes was allowed for this subtest but not for the others. In the test phase, the participants were told to choose between two (one correct and one incorrect) sentence describing a new or old picture.

The scores for each LLAMA subtest range from 0 to 100.

LLAMA test scores have been found to correlate well with learning various aspects of a second language (Abrahamsson & Hyltenstam, 2008; Granena, 2012, 2013; Granena & Long, 2013). The tests have been shown to be internally consistent and test scores are stable over time within participants (Granena, 2013). The scores are unaffected by gender and age for test‐takers above 12 years of age but level of formal education is significantly positively correlated with LLAMA B, E, and F but not D scores (Rogers et al., 2016; Rogers, Meara, Barnett‐Legh, Curry, & Davie, 2017). Hence, we take the LLAMA test battery to be accurate and stable measures of language‐learning aptitude components.

### 2.3 MRI acquisition

MRI was performed with an actively shielded 7T scanner (Achieva, Philips, Best, Netherlands) equipped with a two‐channel transmit and 32 channel receive phased‐array head coil (Nova Medical, Wilmington, MA). Radiofrequency (B1) field inhomogeneities were reduced by using dielectric pads (Teeuwisse, Brink, & Webb, 2012). A T1‐weighted 3D magnetization‐prepared rapid gradient echo (MPRAGE) sequence with repetition time (TR) = 8 ms, echo time (TE) = 1.97 ms, flip angle = 8°, inversion time (TI) = 1,200 ms, outer‐loop SENSE‐factor = 2.5, 0.8 × 0.8 × 0.8 mm^3^ voxels with a scan time of 5:13 min was acquired. For normalization, a proton density (PD)‐weighted gradient echo sequence at identical TR and TE = 1.97 but lower flip angle (2°) and resolution (1.6 × 1.6 × 1.6 mm^3^) with a scan time of 54 s was also acquired (Olsson & Helms, 2021).

Two diffusion‐weighted image volumes were acquired. One single‐shelled with a higher angular resolution, henceforth called the tractography volume, and one multi‐shelled, hereafter the DKI volume, to permit for DKI estimation. The tractography volume was acquired with TR = 9.6 s, TE = 73 ms, flip angle = 90°, SENSE factor = 1.5, partial Fourier = 0.642, 2 × 2 × 2 mm^3^ voxels, 56 directions and *b* = [0 2,000] s/mm^2^ giving a scan time of 9:27 min. Additionally, one extra *b* = 0 volume with a flipped phase encoding direction was acquired for correcting susceptibility induced distortions. The DKI volume was acquired with TR = 6.5 s, TE = 88 ms, flip angle = 90°, SENSE factor = 2, partial Fourier = 0.75, 2 × 2 × 4 mm^3^ voxels, *b* = [0 100 700 1,400 2,000] s/mm^2^ and [1 6 6 12 16] encoding directions giving a scan time of 4:40 min. The ordering of the encoding strengths were randomized in order to temporally spread the energy consumption and avoid temporal confounds (Hutter et al., 2018; Vos et al., 2017).

The collected MRI data is available in OpenNeuro Dataset ds003508 (Novén et al., 2021).

### 2.4 Cortical morphometry

For each participant, the PD‐weighted reference volume was rigidly registered to the T1‐weighted volume using the FMRIB's Linear Image Registration Tool (Jenkinson, Bannister, Brady, & Smith, 2002; Jenkinson & Smith, 2001). Subsequently, the T1‐weighted image was divided by the PD‐weighted reference to mitigate B1 inhomogeneities and eliminate the influence of PD‐weighting and effective transverse (T2*) relaxation. This approach is based on the concept described in Van de Moortele et al. (2009). These volumes were brain extracted using the Brain Extraction Tool (BET) in FSL (Smith, 2002), quality assured and manually edited when needed to ensure that as little nonbrain tissue as possible was included in the segmentation. The normalized and brain‐extracted T1‐weighted image volumes then underwent nonparametric nonuniform bias field correction, to remove residual influence of B1 inhomogeneities (version 2.1.0.post685‐g86e09; Tustison et al., 2010).

Cortical reconstruction and volumetric segmentation was performed with the FreeSurfer image analysis suite (v 6.0), which is documented and freely available for download online (http://surfer.nmr.mgh.harvard.edu/). The technical details of the analysis pipelines and tools for generating aligned maps of cortical thickness and surface area measures have been described elsewhere (Dale, Fischl, & Sereno, 1999; Desikan et al., 2006; Destrieux, Fischl, Dale, & Halgren, 2010; Fischl, Sereno, & Dale, 1999; Fischl et al., 2004; Greve & Fischl, 2009) but a brief summary of and departures from standard analyses follows below.

**FIGURE 1:**
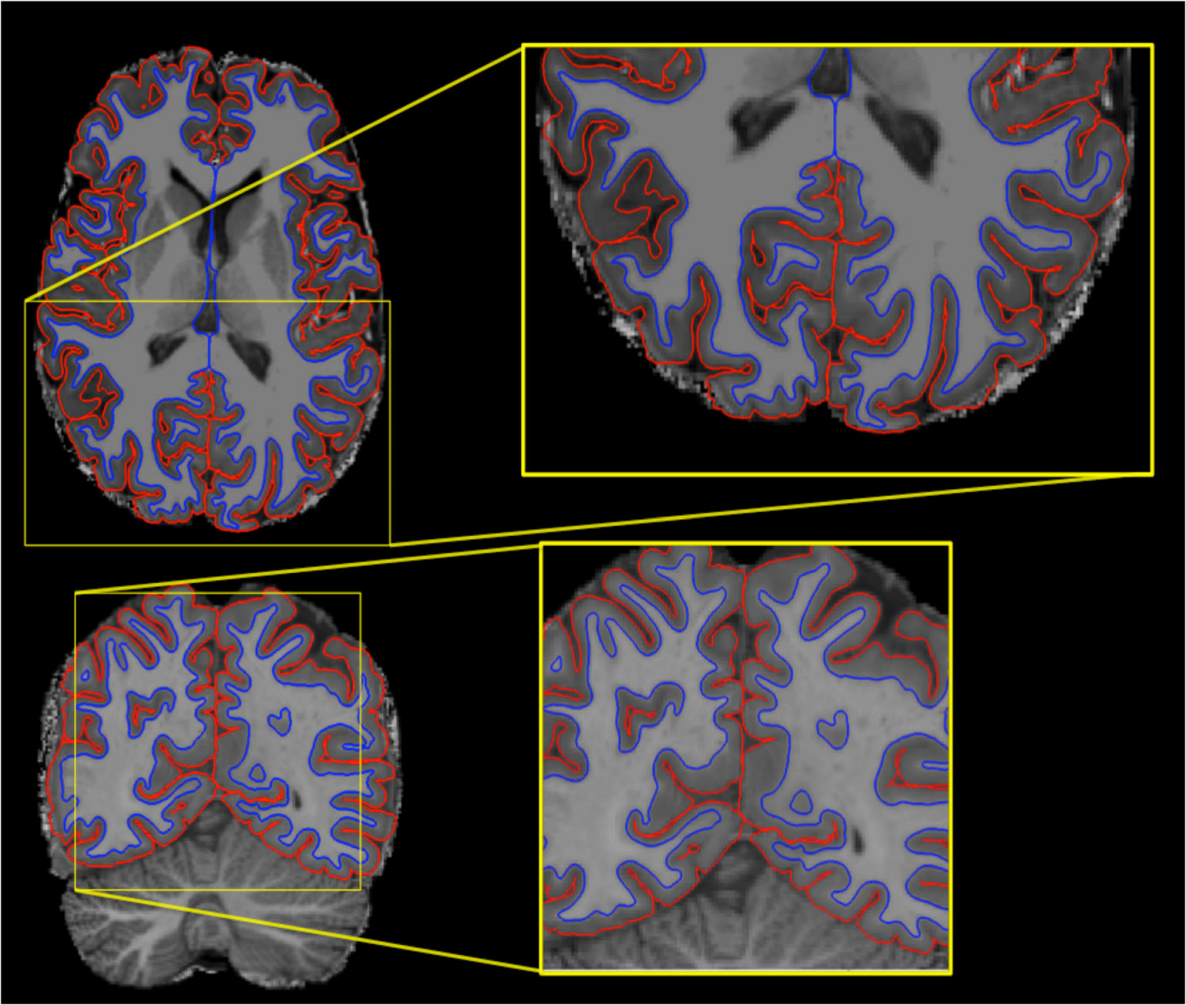
Example of the resulting FreeSurfer surface reconstructions. Pial surfaces are shown in red and white surfaces in blue

The standard recon‐all pipeline was run with the –hires flag (Zaretskaya et al., 2018), except for the skull‐stripping performed as described above. The processing pipeline included motion‐correction, intensity normalization, and tessellation and refinement of the white/grey matter border, from now on called the white surface, and grey/cerebrospinal fluid border, henceforth the pial surface. Cortical surface area is calculated as the area of the tessellation triangles and mapped onto the vertices as the mean of the triangles the vertex is part of. Cortical thickness was computed as the shortest distance between points on the white surface to the pial surface. Maps of cortical thickness and surface area were mapped onto the FreeSurfer common space, FsAverage, and smoothed using a 15 mm full width at half maximum (FWHM) Gaussian kernel. All segmentations and surface reconstructions were visually inspected and manually corrected as needed but the output from one participant was deemed too poor and was left out of further analysis. An example of the resulting surface reconstructions is shown in Figure 1.

### 2.5 DWI processing

The flipped phase encoding volume was used to correct the tractography volume from susceptibility‐induced errors using the topup tool in FSL (Andersson, Skare, & Ashburner, 2003; Smith et al., 2004). At this stage, the tractography volume was corrected for eddy currents and subject motion using ElastiX with extrapolated target volumes (Nilsson, Szczepankiewicz, van Westen, & Hansson, 2015). A first estimation of FA was taken from the TractSeg tool calc_FA and the parameter map was used to register the tractography volume to MNI space using an FA‐template included in FSL. White matter bundle segmentation was performed on the preprocessed tractography volume using the TractSeg tool openly available at https://github.com/MIC-DKFZ/TractSeg (Wasserthal et al., 2018a, 2018b; Wasserthal et al., 2019). TractSeg uses a fully convolutional neural network trained on a cohort from the Human Connectome Project (Van Essen et al., 2013) to automatically segment well‐known anatomical tracts. This is done based on fiber orientation distribution functions obtained from constrained spherical deconvolution (Tournier, Calamante, & Connelly, 2007). The chosen tract segmentations were manually inspected to ensure reasonable segmentation performance. The tractography volume could not be acquired from two participants due to problems with the scanner and data from one participant was discarded from further analysis due to signal loss in the tractography volume in temporal and inferofrontal areas impacting the quality of the segmentations.

The DKI volume underwent correction for noise, Gibbs ringing, Rician biases, and signal outliers followed by motion and eddy current correction identically as for the tractography volume, described above. Thereafter, the DKI volume was smoothed using a 2.4 mm FWHM Gaussian kernel and MD, FA, AD, RD, MK, AK, and RK were estimated using the freely available DESIGNER tool (https://github.com/NYU-DiffusionMRI/DESIGNER; Ades‐Aron et al., 2018) and registered to MNI space. The mean parameter values of each tract were then extracted using the Tractometry command in TractSeg. Parameters from IFOF were only obtainable from 10 of the subjects. Therefore, IFOF was not included in the analyses.

### 2.6 Statistical analyses

To assess the risk that differences in LLAMA test scores reflect differences in other cognitive capacities or linguistic background, we investigated correlations between working memory, fluid intelligence, musicality, or number of learned languages and LLAMA test scores. Such correlations would limit the interpretation of correlations between LLAMA scores and cortical morphometry or tissue microstructure. Moreover, the LLAMA subtests are supposed to measure independent components of language‐learning aptitude and this should be verified. Therefore, the interdependence of LLAMA scores as well as correlations with working memory, general intelligence, musicality, and number of learned languages was assessed using Spearman's rank correlation analyses.

Age and gender were covariates of no interest in all cortical and diffusion parameter analyses. The FreeSurfer tool qdec was used for correlation analyses of cortical thickness and surface area measures. Brain size correlates with cortical thickness and surface area (Im et al., 2008). Therefore, estimated intracranial volume (eICV), as given by FreeSurfer, was included as a covariate of no interest in all cortical thickness and surface area correlation tests. Clusters in which cortical thickness or surface area correlated with a LLAMA test score underwent comparison to cluster size limits derived from Monte Carlo simulation (6,000 permutations)‐based multiple comparison correction (Hagler, Saygin, & Sereno, 2006) using a cluster‐forming threshold of *p* <.005 and *p* <.001 for thickness and surface area, respectively, in accordance with suggestions in Greve and Fischl (2018). Assuming that a cluster‐wise corrected *p*‐value of <.05 is desired, the cluster size limits are 306 and 212 mm^2^ for a cluster forming threshold of *p* <.005 for the left and right hemisphere, respectively. For a cluster‐forming threshold of *p* <.001, the corresponding sizes are 160 and 137 mm^2^. Results were projected onto the pial surface of FsAverage for display purposes. All other statistical analyses were performed using R (R Core Team, 2018). Tables over cortical thickness and surface area for language‐related cortical areas and their correlation statistics (Pearson's correlation with eICV and age as covariates) are provided as Supporting Information. The significance threshold from diffusion parameter correlation tests was Bonferroni corrected for multiple comparisons across the four analyzed white matter tracts, yielding a significance threshold of *p* = .05/4 = 0.0125.

## 3 RESULTS

### 3.1 LLAMA score interdependence

LLAMA score interdependence and dependence on working memory, fluid intelligence, musicality, and number of learnt languages is presented in Table 1, Spearman's *ρ* and uncorrected p‐values. Results showed a weak correlation between LLAMA F and fluid intelligence (Raven score) and a stronger correlation between RAVEN score and number of learned languages. Taken together, the results provided evidence that differences in LLAMA tests within the group of participants did not reflect differences in other cognitive capacities and that LLAMA scores did not correlate with each other, that is, that they captured separate components of language‐learning aptitude. Boxplots of the LLAMA scores are included in Supporting Information.

**TABLE 1:** Spearman's rho and uncorrected *p*‐values for comparisons between each background measure

	LLAMA B	LLAMA D	LLAMA F	OSPAN	Raven	Musicality general sophistication	Number of learned languages
LLAMA B (vocabulary)	1	*ρ* = −0.15 *p* = .27	*ρ* = −0.11 *p* = .40	*ρ* = 0.25 *p* = .062	*ρ* = 0.01 *p* = .94	*ρ* = 0.12 *p* = .38	*ρ* = −0.13 *p* = .34
LLAMA D (phonetics)		1	*ρ* = −0.09 *p* = .51	*ρ* = 0.09 *p* = .51	*ρ* = 0.12 *p* = .38	*ρ* = −0.08 *p* = .54	*ρ* = 0.12 *p* = .38
LLAMA F (grammar)			1	*ρ* = −0.11 *p* = .41	*ρ* = 0.29 *p* = .032*	*ρ* = 0.04 *p* = .78	*ρ* = 0.19 *p* = .15
OSPAN				1	*ρ* = 0.17 *p* = .22	*ρ* = 0.19 *p* = .16	*ρ* = 0.28 *p* = .034*
Raven					1	*ρ* = 0.00 *p* = .98	*ρ* = 0.36 *p* = .0063**
Musicality general sophistication						1	*ρ* = 0.06 *p* = .63
Number of learned languages							1

*Note*: Dark cells are nonsignificant.

* *p* <.05.

** *p* <.01.

### 3.2 White matter tract segmentations

Mean values with standard deviations for all diffusion parameters are presented in Table 2. MD, RD, FA, MK, and RK have been shown to vary between 0.80–0.93 μm^2^/ms, 0.31–0.70 μm^2^/ms, 0.41–0.83, 0.81–1.32, and 1.02–2.54, respectively, in healthy human white matter of the brain (Lätt et al., 2013). While parameter values were comparable to previous estimates in healthy human brains in AF and SLF III, values for UF were unfeasible for white matter tracts. Hence, values from UF were excluded from further analyses.

**TABLE 2:** Mean diffusion parameter values for each studied white matter tract

Diffusion parameter	MD	FA	AD	RD	MK	AK	RK
Left AF	0.89 ± 0.03	0.36 ± 0.02	1.23 ± 0.04	0.72 ± 0.04	1.14 ± 0.04	0.93 ± 0.03	1.41 ± 0.08
Right AF	0.88 ± 0.03	0.36 ± 0.03	1.21 ± 0.03	0.71 ± 0.04	1.12 ± 0.05	0.95 ± 0.04	1.33 ± 0.10
Left SLF III	0.88 ± 0.03	0.38 ± 0.02	1.23 ± 0.03	0.71 ± 0.04	1.13 ± 0.05	0.91 ± 0.04	1.38 ± 0.08
Right SLF III	0.89 ± 0.03	0.36 ± 0.02	1.22 ± 0.03	0.72 ± 0.03	1.12 ± 0.05	0.94 ± 0.04	1.35 ± 0.11
Left UF	1.26 ± 0.24	0.22 ± 0.04	1.57 ± 0.29	1.10 ± 0.20	0.71 ± 0.11	0.76 ± 0.12	0.66 ± 0.11
Right UF	1.11 ± 0.12	0.23 ± 0.03	1.38 ± 0.14	0.97 ± 0.09	0.66 ± 0.06	0.70 ± 0.06	0.63 ± 0.10

*Note*: Mean diffusivity (MD), axial diffusivity (AD), and radial diffusivity (RD) are given in μm^2^/ms while fractional anisotropy (FA), mean kurtosis (MK), axial kurtosis (AK), and radial kurtosis (RK) are dimensionless.

### 3.3 LLAMA B

**FIGURE 2:**
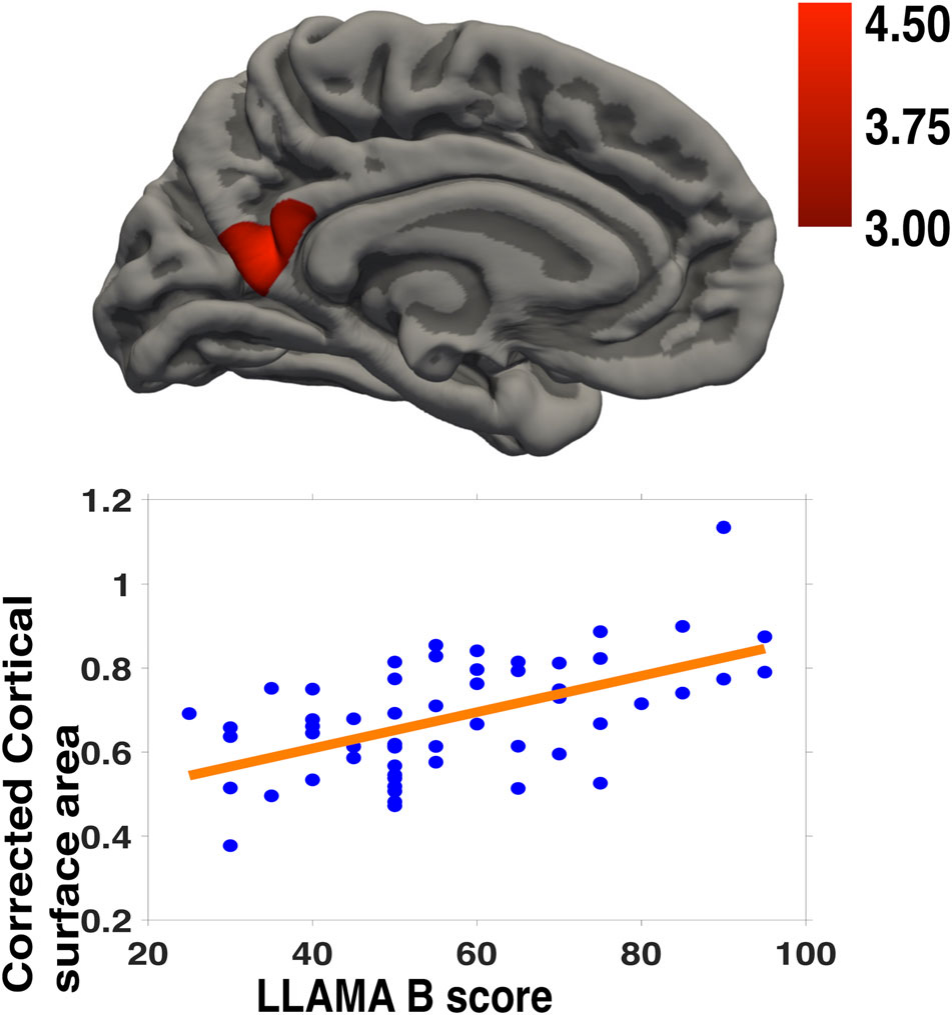
Vocabulary learning aptitude score (LLAMA B) correlated with cortical surface area in a left posterior medial cluster covering the left inferior posterior precuneus, given eICV and age as covariates

Cortical surface area in the left posterior inferior precuneus correlated with LLAMA B scores, as shown in Table 3 and Figure 2. The cluster‐wise *p* in Table 3 is the corrected *p*‐value after correction for multiple comparisons and is specific to the cluster size. No correlations were found between any diffusion parameter in any tract and the LLAMA B score (see Supporting Information for more information).

**TABLE 3:** Statistics for cluster in left posterior inferior precuneus in which cortical surface area correlated with LLAMA B score

Cortical areas	Cluster size (mm^2^)	Pearson's *r*	Peak *Z*	Peak *Z* coordinates (MNI *x*, *y*, *z*)	Cluster‐wise p
L. Precuneus	433	.473	5.11	−7, −59, 13	0.00200

### 3.4 LLAMA D

**FIGURE 3:**
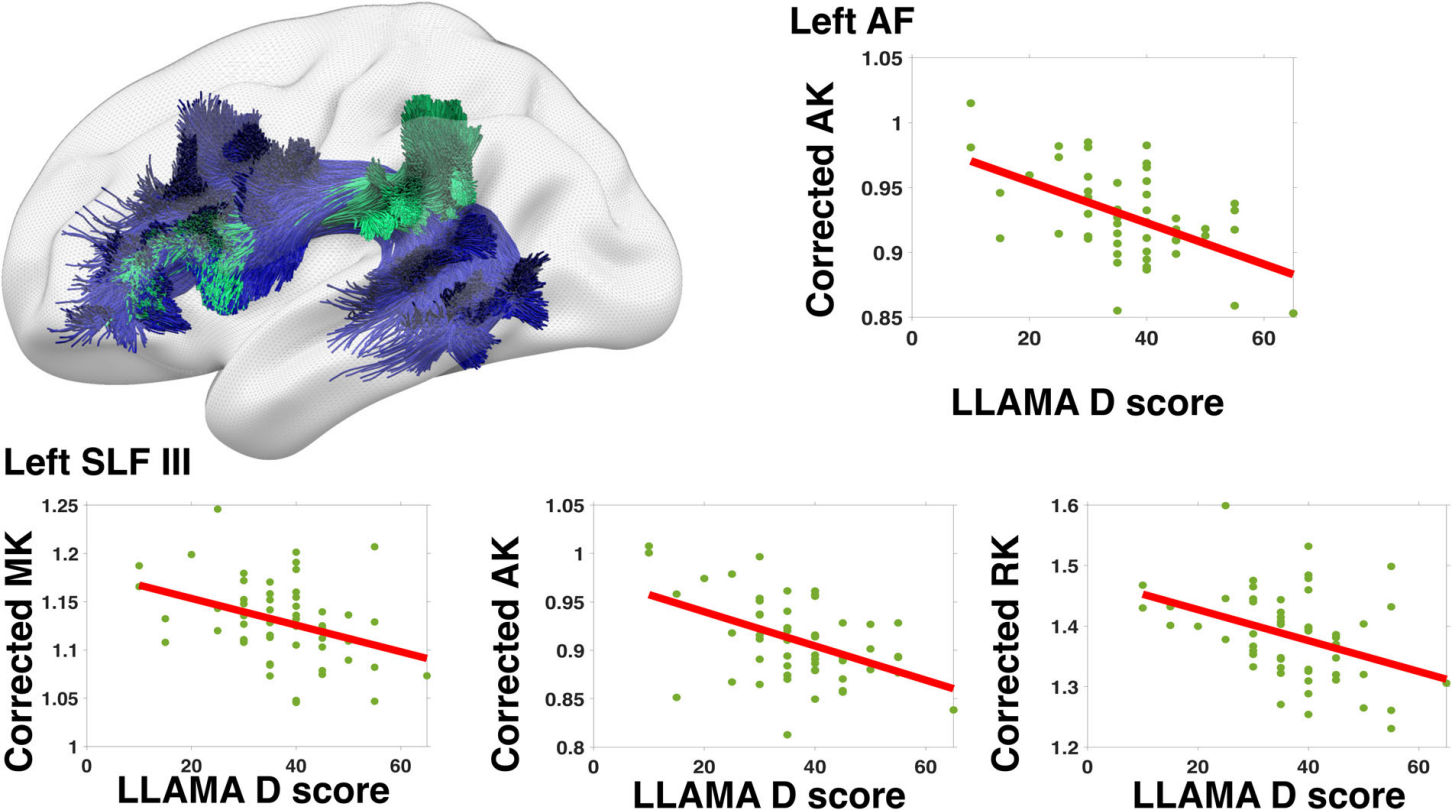
Phonetic memory aptitude (LLAMA D) correlated with axial kurtosis (AK) in left arcuate fasciculus (AF) and left superior longitudinal fasciculus part III. AF (blue) and SLF III (green) are shown in the image to the left, created using SurfIce (freely available at https://github.com/neurolabusc/surf‐ice)

Mean AK along the left AF as well as mean MK, AK, and RK along the left SLF III correlated with phonetic memory (LLAMA D) score (Figure 3 and Table 4). This indicates that greater working memory capacity is associated with lower AK in the white matter tracts that are part of the dorsal language‐processing stream. No correlations were found for the other tracts (see Supporting Information for details).

**TABLE 4:** Correlation coefficients, *t*‐ and uncorrected *p*‐values for significant correlations between diffusion kurtosis parameters and phonetic memory aptitude (LLAMA D)

White matter tract	Diffusion parameter	Pearson's *r*	*t*(51)	*p*
L. AF	AK	−.518	−4.277	8.53e−05
L. SLF III	MK	−.356	−2.696	.0095
	AK	−.440	−3.462	.0011
	RK	−.378	−2.883	.0058

*Note*: Bonferroni‐corrected significance threshold is *p* <.0125.

### 3.5 LLAMA F

No correlations were found between cortical thickness, cortical surface area, or any diffusion parameter in any tract and LLAMA F score (see Supporting Information for more details).

## 4 DISCUSSION

This study investigated cortical morphometric and white matter fiber tract diffusion parameter correlations of language‐learning aptitude. By finding neural foundations for language‐learning abilities, we learn more about how the brain's anatomy can reflect differences in cognitive abilities. We found that greater vocabulary learning aptitude is associated with a larger cortical surface area in a left posterior medial cluster, possibly related to declarative memory capacity (Cavanna & Trimble, 2006). Moreover, phonetic memory negatively correlated with AK in left AF and SLF III, as well as MK and RK in the left SLF III. A negative correlation with MK, AK, and RK can be thought of as phonetic memory benefitting from a more coherent and homogeneous nerve fiber tract. Our results corroborate some and contradict other previous findings. We did not find any correlations between grey matter morphology in right HG (referred to as transverse temporal gyrus in our Supporting Information) and LLAMA scores as expected from Turker et al. (2019). This could be due to the age differences between the groups investigated, as Turker et al. (2019) studied children between 10 and 16 years of age whereas our participants ranged between 20 and 27 years of age. This further suggests that the cortical drivers of language‐learning aptitude are different in different stages of development. Also, while Turker and colleagues solely investigated HG, we performed a whole‐brain analysis. If we assume a coarse parcellation of the cortex into 58 unique areas, used in one of the FreeSurfer parcellations that include HG (Desikan et al., 2006), then the Bonferroni corrected alpha‐threshold for whole cortex analysis would be 0.05/58 ≈ 0.000862. For the correlations between grey matter volume in the right HG and LLAMA subtest scores, reported *p*‐values range from .003 to .028. It should be noted that we found a nonsignificant correlation between cortical thickness in the right HG and LLAMA F score (*r* = .35, uncorrected *p* = .012). See Supporting information for more details. Thus, the correlational results from Turker et al. (2019) would not survive correction for comparison across the entire brain surface. Taken together, the results found in the present study support the claim that individual differences in language‐related performance relate to individual differences in brain structure (Golestani, 2012).

### 4.1 Vocabulary learning aptitude correlates with cortical surface area in left inferior posterior precuneus

The vocabulary learning aptitude LLAMA subtest (LLAMA B) requires the test‐taker to memorise the association of written words with cartoon figures. We found a correlation between this vocabulary learning aptitude score and the cortical surface area in a cluster in the left posterior inferior medial cortex referred to as the inferior posterior part of the precuneus.

The precuneus is involved in the integration between visuospatial inputs and declarative memory (Cavanna & Trimble, 2006), an essential step for LLAMA B test performance. The posterior precuneus is activated more when objects are correctly identified and connected to the correct source memory in episodic memory tests (Lundstrom, Ingvar, & Petersson, 2005). Lundstrom et al. (2005) used a learning task in which participants were presented with a written noun and either a corresponding image or not. Concerning our results, this could indicate that those with high LLAMA B scores use of an episodic component to assist vocabulary learning and that vocabulary learning aptitude in part reflects this cognitive strategy. Left precuneus has a different cortical developmental trajectory in bilingual children and young adults, linked to, in part, increased demands on vocabulary knowledge (Pliatsikas et al., 2020). More left precuneal activity is elicited by remembered than forgotten words 1 week after practicing by naïve learners of Swahili in a vocabulary learning experiment (van den Broek, Takashima, Segers, Fernández, & Verhoeven, 2013). In sum, our results indicate that the cortical surface area of the left inferior posterior precuneus is of importance as regards the aptitude for learning new vocabulary. Relatively larger cortical surface area could speculatively indicate a greater predisposition for storing word‐figure associations.

We did not find support for a correlation between FA in SLF III, arguably the closest correspondence to the tract between BA47 and the parietal lobe reported in (Xiang et al., 2012). This could be explained by Xiang et al.' (2012) use of probabilistic tractography on seed regions derived from functional connectivity patterns of the components of Broca's area (Xiang, Fonteijn, Norris, & Hagoort, 2010). We instead chose to use an automatic segmentation tool to ensure the anatomical validity of the included tracts.

### 4.2 Phonetic memory benefits from a coherent and more homogeneous left AF and SLF III

Phonetic memory capacity (LLAMA D) score correlated negatively with AK in the left AF and SLF III as well as MK and RK the left SLF III. The AF connects the IFG with the middle frontal gyrus, the posterior superior temporal gyrus and the temporal occipital transition region, while the adjacent SLF III connects the supramarginal gyrus with the prefrontal and ventral premotor cortices. AK and RK are indexes of tissue complexity along and across the principal diffusion direction, that is, along the fibers in an ideal white‐matter voxel, respectively. MK is the average apparent kurtosis along all diffusion encoding directions. Higher AK could be due to the presence of nonaxonal cell membranes, for example, glial cells, astrocytes, and oligodendrocytes (Hui, Cheung, Qi, & Wu, 2008) or tortuosity of the axons (Fieremans, Jensen, & Helpern, 2011). Higher RK could additionally mean differences in axonal radii (Fieremans et al., 2011). While it is important to note that the DKI parameters are nonspecific to microstructural features, an intuitive interpretation of the results would be that more coherent and more homogeneous left AF and SLF III are beneficial for phonetic memory. This would hold for the tissue complexity in the left SLF III overall, not just in the principal fiber direction. Both left AF and SLF III are part of the dorsal language processing stream and are involved in the repetition of especially pseudowords (Hickok & Poeppel, 2004; Saur et al., 2008).

### 4.3 Perceptiveness to and learning foreign speech sounds

The ability to repeat meaningless words could perhaps be correlated with the implicit memory for foreign speech sounds as both, to some extent, require perceiving and temporarily storing novel sounds. It has been shown that expert phoneticians have larger pars opercularis of the left IFG, connected to the AF, and higher probability of split as well as more white matter in HG, bilaterally (Golestani et al., 2011). Golestani et al. did not investigate the white‐matter connections between these areas but the implicit connection between the temporal lobe and the inferior frontal cortex fits our results well, assuming that trained phoneticians have greater phonetic memory capacity. Moreover, learners who are faster at perceiving novel speech sound contrasts have more white matter in (especially left) parietal regions (Golestani, Paus, & Zatorre, 2002). Success in learning Mandarin Chinese words from listening to a short film has been seen to be associated with greater functional connectivity between the left supplementary motor area and precentral gyrus as well as the left insula and the left rolandic operculum (Veroude, Norris, Shumskaya, Gullberg, & Indefrey, 2010). The structural connection between the supplementary motor area and precentral gyrus is part of SLF III and our results correspond well with the suggested importance of the functional connection for phonological rehearsal, but not necessarily speech articulation. Taken together, our findings are well in accordance with previously reported associations between the morphology of cortical areas connected by the left AF and SLF III and perceptiveness and learning aptitude for foreign speech sounds.

### 4.4 Phonological working memory

Studies of neural correlates of phonological working memory have directly implicated cortical areas connected by the left AF and SLF III, fitting well with the results presented here. Active phonological working memory tasks activate left IFG and posterior superior temporal sulcus (STS) in a “maintenance” phase and bilateral intraparietal sulcus, IFG and STS during the “comparison and decision” phase (Strand, Forssberg, Klingberg, & Norrelgen, 2008). The task used in Strand et al. (2008) differed from LLAMA D in that subjects were asked to actively keep the stimulus syllable strings in memory. Phonological working memory load is correlated with recruitment of bilateral superior temporal gyrus, left planum temporale and left precentral gyrus (Scott & Perrachione, 2019). More specifically, the posterior‐medial planum temporale has been suggested to be a critical region for phonological working memory, supposedly as a site for “phonological storage” (McGettigan et al., 2010). Patients suffering from conduction aphasia, strongly associated with damage of the left AF (Damasio & Damasio, 1980; Tanabe et al., 1987), typically exhibit impaired phonological memory (Bartha & Benke, 2003). Altogether, left AF and SLF III connect important cortical areas for phonological working memory and our results indicate the importance for coherency and homogeneity of the white matter tracts for the ability to implicitly learn foreign speech sounds.

### 4.5 Lack of correlations with LLAMA F

We did not replicate the findings in (Novén et al., 2019) that LLAMA F score correlates with cortical thickness in the left IFG pars triangularis and rostral middle frontal gyrus. In fact, we found no correlations at all with cortical thickness in the present study; a table with mean values of cortical thickness and surface area in several language‐relevant cortical areas are provided as Supporting Information. One important difference between this and our previous study is that the T1‐weighted images are of higher resolution (0.8 mm isotropic versus 1 mm isotropic) and thereby have less partial volume effects. This study included a greater number of participants (54 as opposed to 32). While this increases statistical power and thus increases the likelihood of finding associations, it is possible that this sample of subjects included more participants prone to use a strategy that does not benefit from greater cortical thickness in the aforementioned areas. The ratio of participants that scored more than 75 on the LLAMA F subtest, a score considered unusually high (Meara, 2005), was greater in Novén et al. (2019) 46.8% compared to 31.5% in this study. However, the LLAMA F test scores did not differ significantly (*t*[62.8] = −0.932, *p* = .355, Welch two‐sample *t*‐test) between this study (M = 63.5, *SD* = 19.7) and Novén et al. (2019) (M = 67.9, *SD* = 22.8). Future studies could include a post‐test survey to identify if there are differences in strategies to solve the task between subjects. Xiang et al. (2012) found correlations between grammatical inferencing aptitude and left‐lateralization of the connection between BA45 and the posterior temporal lobe as well as the sum of the number of streamlines between BA6 and the posterior temporal lobe from both hemispheres. We found no correlations between any diffusion parameters in AF, arguably the closest corresponding tract in this study, and grammatical inferencing aptitude scores. This might, again, be due to differences in white‐matter segmentation strategies.

Grammatical inferencing aptitude correlated with fluid intelligence but not with working memory, musicality, or number of learned languages. This replicates a known association between grammatical inferencing and intelligence (Cox, Lynch, Mendes, & Zhai, 2019). No other language‐learning aptitude score correlated with any background or cognitive measure. This provides evidence that the LLAMA test battery measures language‐learning specific cognitive constructs, not dependent on other aspects of cognitive capacity except for a moderate link between fluid intelligence and grammatical inferencing score.

### 4.6 Limitations and future directions

We identified five primary types of limitations in the present study. First, we did the imaging at an ultra‐high field strength of 7T. While the ultra‐high field generally generates higher SNR and CNR, the magnetic field is also less homogeneous and susceptibility artifacts are more pronounced (van der Kolk, Hendrikse, Zwanenburg, Visser, & Luijten, 2013). However, we made substantial efforts to mitigate the effects of field inhomogeneities to make the results more reliable. FreeSurfer performs well at higher resolution but thickness estimates tend to decrease except in the cingulate and calcarine sulci as well as in the posterior bank of the central sulcus (Zaretskaya et al., 2018). The mitigated partial volume effects should improve the cortical thickness and surface area estimations. Maps of the mean and standard deviations of cortical thickness estimates from the data in this study and Novén et al. (2019) are given as Supporting Information. The present results should thus be more reliable than results from data with lower resolution.

The second limitation concerns the white matter segmentation. The TractSeg tool we used for this purpose was trained on data from lower field‐strength MRI. However, a visual inspection of the tract segmentations indicates good performance on the 7T data for AF and SLF III (Figure 3). Still, the segmentation of IFOF did not map well to the DKI volume and diffusion parameters from UF were unfeasible and thus excluded. This is probably due to imaging artifacts in the inferior part of the DKI images. There could, therefore, exist correlations between the tissue microstructure of these tracts and language‐learning aptitude scores to be discovered in future studies using methods that mitigate these issues. The slightly lower mean FA values found for AF and SLF III in this study probably have to do with the TractSeg segmentations being more generous than the manually positioned ROIs in Lätt et al. (2013).

The third limitation is that the LLAMA tests' ability to capture neuro‐relevant variance depends on how well they match the granularity of the role of the cortical area/fiber tract. Future work could fine‐tune the language‐learning aptitude tests to divide the tests into components that match the functional roles of the different cortical areas. The validity of such tests must be motivated by functional studies (not necessarily fMRI). Nevertheless structural MRI could continue to capture brain morphological differences due to heightened acuity/aptitude for what the component captures.

The fourth limitation is that this study could potentially be limited in statistical power. This could possibly explain the few significant correlations found relative to the number of tests performed. A reasonable estimate (based on the studies cited in this article) of the correlation coefficients we can expect in correlations between behavioral measures and diffusion parameters or cortical morphometrical measures range from *r* = .35–.56. Given a significance threshold of .05, we thus need between 23 and 62 participants to detect such correlation at a power of .8. This is assuming only one ROI but it is normal to include at least the contralateral homologue of the ROI in neuroimaging studies. If the significance threshold is Bonferroni corrected according to the diffusion analysis in our study with a reasonable number of ROIs (0.05/4), then the number of subjects needs to increase to between 31 and 86. For cortical morphometry, the measures range from *r* = .46 to .66 but have to be corrected for at least 58 ROIs (see reasoning above), requiring a number of subjects between 31 and 73. As we included 57 subjects, we are confident that our results, although validation is necessary, can increase our understanding of the neural underpinnings of language‐learning aptitude value to the field. However, future studies could benefit from including more subjects.

The fifth limitation is that we only studied young adult subjects and that the white matter maturation might not be complete. Studies have found both DTI parameters (Chen, Zhang, Yushkevich, Liu, & Beaulieu, 2016) and MK (Falangola et al., 2008) to be relatively stable in early adults. However, the MRI techniques we deploy might lack sensitivity regarding differences in white matter maturation.

## 5 CONCLUSIONS

In conclusion, our results suggested a benefit from larger cortical surface in a left posterior medial cluster for vocabulary learning aptitude, possibly reflecting a greater declarative memory storing capacity for linguistically relevant word‐figure associations. Moreover, the kurtosis parameters in known dorsal language processing stream tracts correlated negatively with phonetic memory. This indicates an impact from the coherency and homogeneity of white matter tracts connecting well‐known cortical areas responsible for phonological storage on phonetic working memory. Our findings add to the knowledge of how cortical thickness, cortical surface area, and tissue microstructure of white matter fiber tracts correlate with talent for learning languages. In a wider perspective, this also means that differences in aptitudes and talents could, at least partly, be due to differences in cortical morphometry or white matter microstructure.

## CONFLICT OF INTEREST

The authors declare no potential conflict of interest.

## Supporting information

**Appendix S1.** Supporting Information.

## Data Availability

The data that support the findings of this study are openly available in " Language Learning Aptitude dataset" at https://openneuro.org/datasets/ds003508, doi: 10.18112/openneuro.ds003508.v1.0.0.
