# Supplementary material for: Cortical and white matter correlates of language‐learning aptitudes
Source: Hum Brain Mapp. 2021 Jul 20;42(15):5037–50. doi: 10.1002/hbm.25598 (PMC8449104; doi:10.1002/hbm.25598)
Supplement: Supplementary file 1 — Appendix S1. Supporting Information. [file HBM-42-5037-s001.pdf]

Supplementary Information

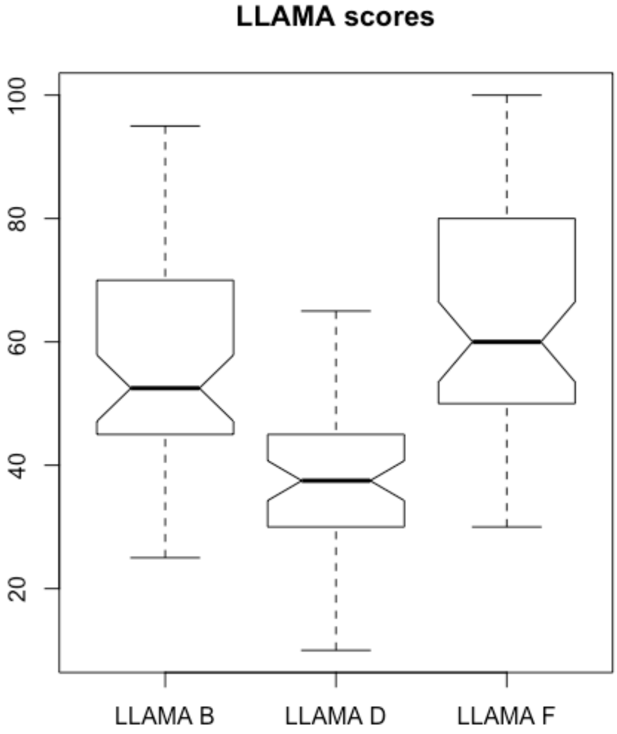

Figure 1: LLAMA scores for all participants. All LLAMA test scores range from 0 to 100.

Correlation tests on cortical thickness and surface area from relevant cortical areas on each LLAMA tests. Age and estimated intracranial volume added as a covariate of no interest to all analyses.

CorticalArea	Mean Cortical Thickness ± SD (mm)	LLAMAB correlation Pearson's r	LLAMAB correlation p	LLAMAD correlation Pearson's r	LLAMAD correlation p	LLAMAF correlation Pearson's r	LLAMAF correlation p
Left Superior Temporal Gyrus	2.30±0.19	0.275	0.046	-0.147	0.293	0.201	0.152
Left Middle Temporal Gyrus	2.27±0.19	0.149	0.287	-0.046	0.742	0.221	0.115
Left Transverse Temporal Gyrus	2.75±0.19	0.106	0.449	0.116	0.407	0.145	0.305
Left Inferior Frontal Gyrus pars opercularis	2.33±0.32	0.132	0.347	-0.304	0.027	0.089	0.531
Left Inferior Frontal Gyrus pars triangularis	2.29±0.30	0.192	0.169	-0.330	0.016	-0.030	0.832
Left Supramarginal	2.73±0.13	0.083	0.557	-0.054	0.702	0.185	0.189
Right Superior Temporal Gyrus	2.66±0.29	-0.019	0.891	0.091	0.517	0.051	0.717
Right Middle Temporal Gyrus	2.29±0.36	-0.074	0.597	0.063	0.655	0.114	0.422
Right Transverse Temporal Gyrus	2.85±0.21	-0.067	0.631	-0.064	0.647	0.346	0.012
Right Inferior Frontal Gyrus pars opercularis	2.64±0.17	-0.110	0.433	-0.003	0.985	0.016	0.908
Right Inferior Frontal Gyrus pars triangularis	2.53±0.20	0.081	0.564	-0.256	0.064	0.028	0.842
Right Supramarginal	2.52±0.20	-0.065	0.643	0.070	0.618	0.097	0.495

CorticalArea	Mean Cortical Surface Area ± SD (mm)	LLAMAB correlation Pearson's r	LLAMAB correlation p	LLAMAD correlation Pearson's r	LLAMAD correlation p	LLAMAF correlation Pearson's r	LLAMAF correlation p
Left Superior Temporal Gyrus	3910±400	0.094	0.503	0.001	0.997	-0.019	0.891
Left Middle Temporal Gyrus	2990±370	0.015	0.913	0.177	0.205	0.338	0.014
Left Transverse Temporal Gyrus	530±90	-0.208	0.136	-0.153	0.275	-0.045	0.753
Left Inferior Frontal Gyrus pars opercularis	1680±240	0.126	0.369	-0.049	0.726	0.087	0.536
Left Inferior Frontal Gyrus pars triangularis	1420±210	0.080	0.571	-0.115	0.411	-0.063	0.657
Left Supramarginal	4140±590	0.094	0.503	0.186	0.184	0.181	0.199
Right Superior Temporal Gyrus	3690±350	0.158	0.259	-0.033	0.813	0.047	0.740
Right Middle Temporal Gyrus	3370±360	-0.114	0.415	0.058	0.678	-0.089	0.529
Right Transverse Temporal Gyrus	420±80	0.191	0.171	0.126	0.368	-0.063	0.654
Right Inferior Frontal Gyrus pars opercularis	1390±220	0.144	0.304	-0.014	0.924	-0.013	0.924
Right Inferior Frontal Gyrus pars triangularis	1580±280	0.094	0.501	0.075	0.591	0.014	0.922
Right Supramarginal	3800±470	0.052	0.710	0.269	0.052	-0.182	0.197

Correlation tests from each diffusion parameter on each LLAMA tests.

Correlation coefficients, t- and p-values from correlation tests from each diffusion parameter with LLAMA B (vocabulary learning aptitude) score.

	Diffusion parameter	MD	FA	AD	RD	MK	AK	RK
Left AF	Pearson's r	0.013	0.127	0.138	-0.045	0.218	0.134	0.161
	t	0.092	0.908	0.987	-0.318	1.580	0.957	1.155
	p	0.927	0.368	0.328	0.752	0.121	0.343	0.253
Right AF	Pearson's r	0.063	-0.064	0.048	0.058	0.109	0.018	0.188
	t	0.446	-0.451	0.338	0.414	0.777	0.130	1.355
	p	0.658	0.654	0.737	0.681	0.441	0.897	0.182
Left SLF III	Pearson's r	0.024	0.114	0.053	-0.056	0.311	0.093	0.160
	t	-0.173	0.814	0.377	-0.394	2.310	0.664	1.148
	p	0.864	0.420	0.708	0.695	0.025	0.510	0.257
Right SLF III	Pearson's r	0.019	-0.139	-0.088	0.067	0.003	-0.156	0.024
	t	0.137	-0.990	-0.622	0.478	0.020	-1.118	0.173
	p	0.892	0.327	0.537	0.635	0.984	0.269	0.863

Correlation coefficients, t- and p-values from correlation tests from each diffusion parameter with LLAMA D (phonetic memory) score.

	Diffusion parameter	MD	FA	AD	RD	MK	AK	RK
Left AF	Pearson's r	0.089	-0.092	0.077	0.084	-0.297	**-0.518**	-0.244
	t	0.631	-0.654	0.546	0.597	-2.199	**-4.277**	-1.783
	p	0.531	0.516	0.588	0.553	0.033	**8.529e-05**	0.081
Right AF	Pearson's r	0.195	-0.138	0.152	0.179	-0.200	-0.266	-0.195
	t	1.403	-0.988	1.087	1.284	-1.445	-1.951	-1.406
	p	0.167	0.328	0.282	0.205	0.155	0.057	0.166
Left SLF III	Pearson's r	0.081	-0.179	0.015	0.100	**-0.356**	**-0.440**	**-0.378**
	t	0.571	-1.287	0.107	0.710	**-2.696**	**-3.462**	**-2.883**
	p	0.570	0.204	0.915	0.481	**0.0095**	**0.0011**	**0.0058**
Right SLF III	Pearson's r	0.092	0.114	0.242	0.000	-0.067	-0.113	-0.071
	t	0.653	0.811	1.766	0.003	-0.471	-0.802	-0.506
	p	0.517	0.421	0.084	0.998	0.639	0.426	0.615

Correlation coefficients, t- and p-values from correlation tests from each diffusion parameter with LLAMA F (grammatical inferencing aptitude) score.

	Diffusion parameter	MD	FA	AD	RD	MK	AK	RK
Left AF	Pearson's r	-0.042	0.071	-0.018	-0.048	-0.039	-0.057	-0.046
	t	-0.297	0.506	-0.126	-0.341	-0.276	-0.403	-0.324
	p	0.768	0.615	0.900	0.735	0.784	0.689	0.747
Right AF	Pearson's r	-0.091	0.069	-0.042	-0.096	-0.023	0.060	-0.150
	t	-0.644	0.491	-0.295	-0.683	-0.160	0.422	-1.075
	p	0.523	0.625	0.769	0.497	0.874	0.675	0.288
Left SLF III	Pearson's r	0.084	0.003	0.081	0.076	0.091	0.156	0.034
	t	0.599	0.023	0.576	0.539	0.648	1.117	0.241
	p	0.552	0.982	0.567	0.593	0.520	0.269	0.810
Right SLF III	Pearson's r	-0.004	0.044	0.039	-0.024	0.032	0.160	-0.105
	t	-0.028	0.312	0.274	-0.169	0.227	1.148	-0.746
	p	0.978	0.757	0.785	0.866	0.821	0.257	0.459

Comparisons between thickness estimates between Novén et al. (2019) and the present study

In our previous work (Novén et al., 2019), we used different MRI sequences for obtaining anatomical MRI images for cortical thickness estimation, see table below. Perhaps of most importance is the difference in spatial resolution (1 mm^3 isotropic versus 0.8 mm^3 in the present study). Figure 1 shows the group mean thickness from both studies while figure 2 shows the standard deviation of the group thickness estimates from each of the studies. Cortical thickness estimates are in general slightly greater in the present study. Standard deviations within the groups seem comparable, indicating that the greater resolution does not entail less varying cortical thickness estimate.

Parameters of the anatomical MRI sequences from Novén et al. (2019) and the present study.

Study	Repetition time (T1 volume)	Echo time (T1 volume)	Flip angle (T1 volume)	Inverstion time (T1 volume)	Repetition time (PD volume)	Echo time (PD volume)	Flip angle (PD volume)	Inverstion time (PD volume)	Resolution of corrected volume
Novén et al. (2019)	5 ms	2 ms	6°	1200 ms	6 ms	2.5 ms	7°	1200 ms	1 mm^3 isotropic
Present	8 ms	1.97 ms	8°	1200 ms	8 ms	1.97 ms	2°	1200 ms	0.8 mm^3 isotropic

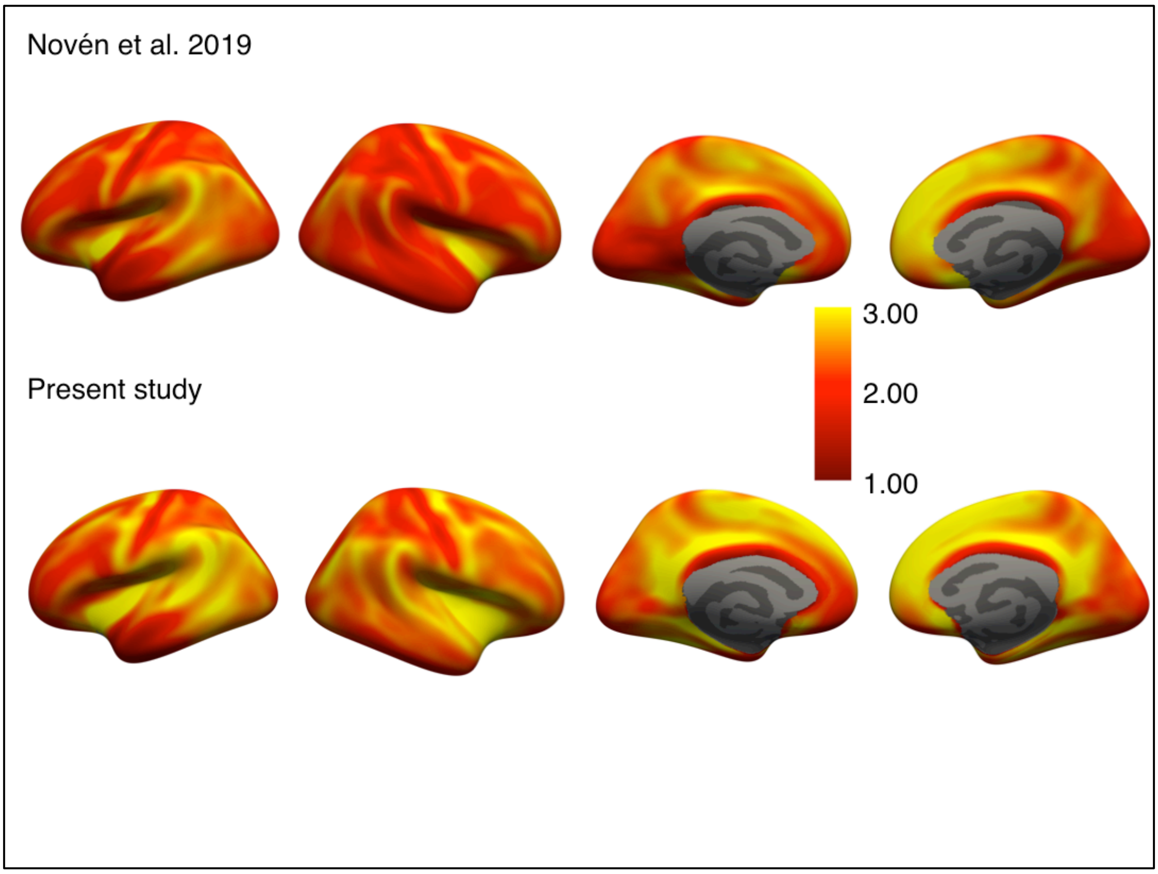

Figure 2: Group mean thickness across the cortex in the two studies.

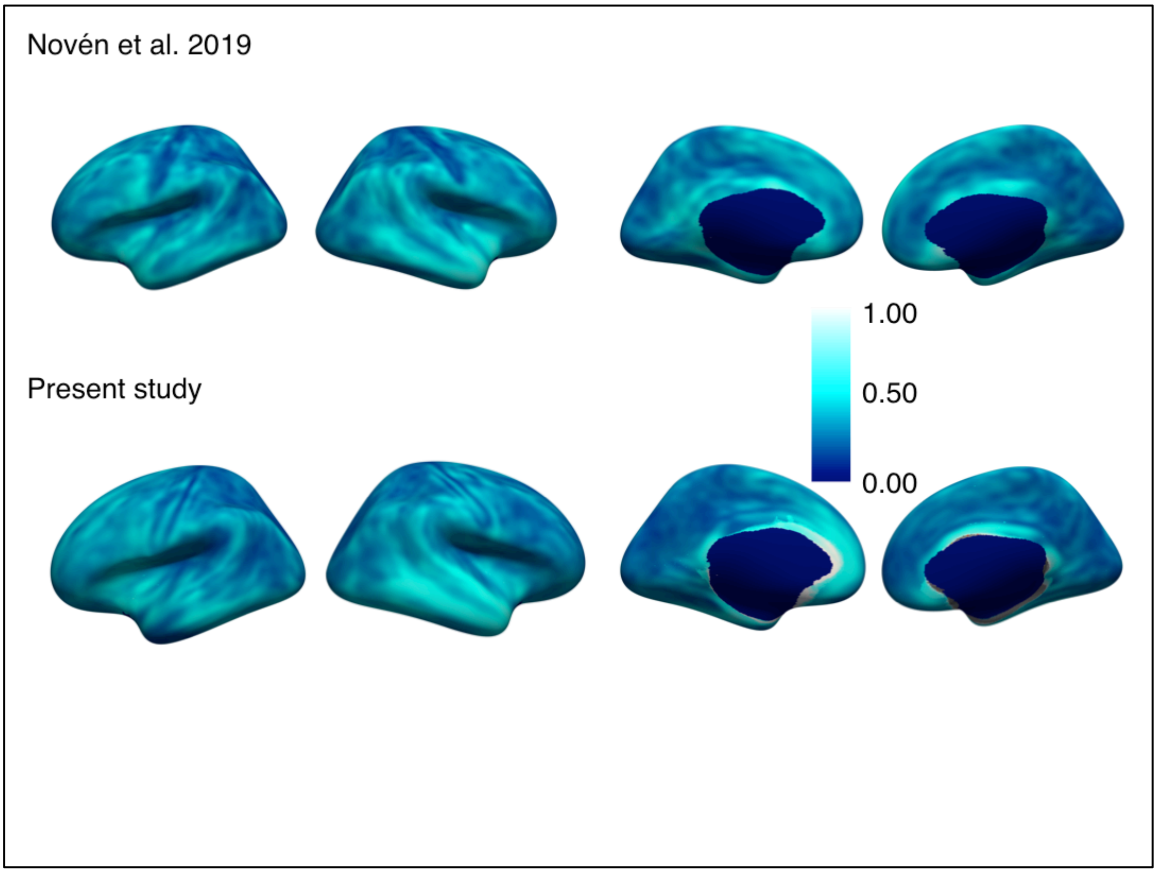

Figure 3: Standard deviations in cortical thickness estimates for the groups in the two studies.

References

Novén M, Schremm A, Nilsson M, Horne M, Roll M (2019) Cortical thickness of Broca's area and right homologue is related to grammar learning aptitude and pitch discrimination proficiency. Brain and Language 188:42-47.
